# CC Chemokine Family Members’ Modulation as a Novel Approach for Treating Central Nervous System and Peripheral Nervous System Injury—A Review of Clinical and Experimental Findings

**DOI:** 10.3390/ijms25073788

**Published:** 2024-03-28

**Authors:** Agata Ciechanowska, Joanna Mika

**Affiliations:** Department of Pain Pharmacology, Maj Institute of Pharmacology Polish Academy of Sciences, 12 Smetna Str., 31-343 Kraków, Poland; ciechan@if-pan.krakow.pl

**Keywords:** chemokines, chemokine receptors, nervous system injury, pain, antagonist

## Abstract

Despite significant progress in modern medicine and pharmacology, damage to the nervous system with various etiologies still poses a challenge to doctors and scientists. Injuries lead to neuroimmunological changes in the central nervous system (CNS), which may result in both secondary damage and the development of tactile and thermal hypersensitivity. In our review, based on the analysis of many experimental and clinical studies, we indicate that the mechanisms occurring both at the level of the brain after direct damage and at the level of the spinal cord after peripheral nerve damage have a common immunological basis. This suggests that there are opportunities for similar pharmacological therapeutic interventions in the damage of various etiologies. Experimental data indicate that after CNS/PNS damage, the levels of 16 among the 28 CC-family chemokines, i.e., CCL1, CCL2, CCL3, CCL4, CCL5, CCL6, CCL7, CCL8, CCL9, CCL11, CCL12, CCL17, CCL19, CCL20, CCL21, and CCL22, increase in the brain and/or spinal cord and have strong proinflammatory and/or pronociceptive effects. According to the available literature data, further investigation is still needed for understanding the role of the remaining chemokines, especially six of them which were found in humans but not in mice/rats, i.e., CCL13, CCL14, CCL15, CCL16, CCL18, and CCL23. Over the past several years, the results of studies in which available pharmacological tools were used indicated that blocking individual receptors, e.g., CCR1 (J113863 and BX513), CCR2 (RS504393, CCX872, INCB3344, and AZ889), CCR3 (SB328437), CCR4 (C021 and AZD-2098), and CCR5 (maraviroc, AZD-5672, and TAK-220), has beneficial effects after damage to both the CNS and PNS. Recently, experimental data have proved that blockades exerted by double antagonists CCR1/3 (UCB 35625) and CCR2/5 (cenicriviroc) have very good anti-inflammatory and antinociceptive effects. In addition, both single (J113863, RS504393, SB328437, C021, and maraviroc) and dual (cenicriviroc) chemokine receptor antagonists enhanced the analgesic effect of opioid drugs. This review will display the evidence that a multidirectional strategy based on the modulation of neuronal–glial–immune interactions can significantly improve the health of patients after CNS and PNS damage by changing the activity of chemokines belonging to the CC family. Moreover, in the case of pain, the combined administration of such antagonists with opioid drugs could reduce therapeutic doses and minimize the risk of complications.

## 1. Introduction

Recent research has shown that comparisons of changes occurring after brain damage, as well as after injury to peripheral nerves, are important because often these changes have a common immunological basis. Importantly, this background may contribute to identifying drugs effective for treating injuries to the central and peripheral nervous systems. This is especially important since traumatic brain injury is one of the leading causes of death and permanent disability worldwide, and its annual global incidence is variable, estimated at 27 to 69 million injured people [1,2]. After such injury, there is direct damage to the brain structures at the site of the impact, which may include contusions and hemorrhages and thus cause loss of consciousness, motor and sensory deficits, and other temporary or permanent neurological symptoms [3]. However, secondary processes are what cause CNS injuries to have various etiologies and are complex issues. These secondary processes include, among others, hypoxic-ischemic injury, altered metabolic and vascular permeability, cerebral edema, diffuse axonal damage, hydrocephalus, and increased intracranial pressure. Ongoing neurodegeneration involves both the gradual loss of neurons and the activation of many cells and contributes to the disruption of neuroplasticity, neuronal networks, signal transmission, and communication between various areas of the brain [3]. However, the activation of glial and immune cells is still not fully understood [4,5]; moreover, apoptosis, necrosis, and axonal degeneration occur, as does the formation of amyloid plaques around neurons [6,7,8]. The abovementioned processes lead to long-term neurodegenerative changes and cognitive disorders, such as memory loss and dementia [9,10,11,12,13]. Because post-traumatic brain pathophysiology develops within a few days after injury, there is a therapeutic window that allows for the possibility of pharmacological intervention. Literature data clearly show that brain damage results in the development of central pain (changes in the second or third sensory neuron). The acute pain sensations that arise are related to a specific dysfunction and usually last up to several weeks [14]. Most of the research on pain hypersensitivity resulting from brain injury concerns the head, which is the most common site of discomfort in patients [15,16]. Other commonly reported sites of pain are the musculoskeletal tract, including the neck, shoulders, back, and limbs [17]. Additionally, many patients with moderate-to-severe CNS damage experience painful spasticity associated with limb stiffness, uncontrolled muscle movements, and poor coordination [18]. Some patients with brain or spinal cord injury develop late-onset pain syndrome with symptoms appearing six or more months after the injury [19]. Finally, all reports clearly show that dysfunctions associated with CNS damage are very heterogeneous in nature and are difficult to treat. Persistent inflammation contributes to neuronal apoptosis and the activation of glial and immune cells, which intensifies pain processes [14,20,21]. Unfortunately, therapeutic strategies for central pain are limited because the pathomechanism is not fully understood.

It is worth noting that treating disorders resulting from damage to peripheral nerves seems to be equally difficult. The published data indicate that peripheral nerve injuries constitute 2 to 3% of all diseases and most often occur during traffic accidents [22] and warfare [23]. Iatrogenic injuries caused by medical procedures account for 17.4% of treated nerve damage [24]. The most common injuries include lacerations, which cause a partial or complete loss of nerve continuity, and compression injuries, which may result in a loss of sensory nerve function despite maintaining complete continuity. This difference is believed to be caused by both ischemia and mechanical deformation as a direct result of compression. Other less common mechanisms include thermal injury or ischemia caused by vascular damage [22,25,26,27]. This damage may lead to a painful response, even in response to nonpainful stimuli [28]. This type of pain is called neuropathic pain and is particularly difficult to treat. Sensitization to sensory neuron stimulation contributes to the release of numerous nociceptive factors, both by neuronal and immune cells at the site of injury [29]. Literature data indicate that among immune cells, neutrophils are the first to react to nerve damage, followed by macrophages and lymphocytes both at the site of damage and in the dorsal root ganglia. However, numerous studies suggest that neutrophils, macrophages, and microglia are significantly involved in the development of central sensitization at the spinal cord level [30,31,32,33,34,35]. Neuropathic pain has a very diverse etiology; therefore, it is a condition that requires multidirectional therapeutic treatment. However, the mechanism of its formation still leaves many questions, and further in-depth research is needed. A growing number of scientific reports suggest that chemokines are key pain mediators [36], which we will discuss in the following sections.

We would like to emphasize that after damage to the CNS and PNS occurs, a number of changes in immunological factors that play important roles in repair and regulatory and inflammatory processes occur. The activation of secondary molecular cascades occurs within a few days after the primary injury, which creates opportunities for pharmacological therapeutic interventions. However, it is necessary to understand the factors underlying these changes. In this review, we will focus on the role of selected CC chemokines and their receptors (Table 1). To date, most of the knowledge about these chemokines came from animal studies. Especially, rodents like mice and rats serve as well-established animal models in research because of their physiological, anatomical, and genetic similarity to humans. Other advantages of using these rodents in science include their size, strain diversity, easy breeding, and, most importantly, the availability of models that reflect CNS and PNS damage occurring in patients. Animal models enable the study of neuroimmune changes in the CNS at both the molecular and cellular levels in controlled conditions and also of using new compounds, which is not possible in clinical trials.

**Scheme 1:**
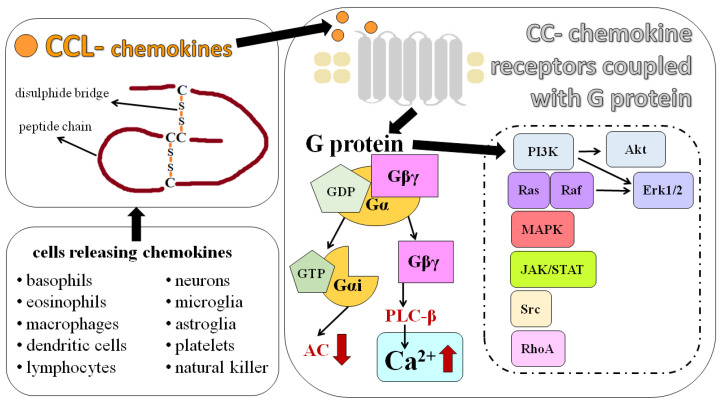
Cells releasing chemokines, chemical structure of chemokines, and intracellular cascades activated by binding of chemokines to their receptors. Abbreviations: GTP, Guanosine triphosphate; GDP, Guanosine diphosphate; Gα, G protein α subunit; Gαi, G protein αi subunit; Gβγ, G protein βγ subunits; PI3K, Phosphoinositide 3-kinase; Akt, Protein kinase B; Ras, Rat sarcoma virus; Raf, Rapidly accelerated fibrosarcoma; Erk1/2, Extracellular signal-regulated kinases; MAPK, Mitogen-activated protein kinase; JAK/STAT, Janus kinase/signal transducers and activators of transcription; Src, Proto-oncogene tyrosine-protein kinase; Rho A, Transforming protein RhoA; AC, adenylate cyclase; PLC-β, Phospholipase C β; PKA, protein kinase A. Scheme created based on data from the following literature [39,41,42,43,44,45,46,47].

The CC group is the largest one among chemokines and is characterized by the presence of the first two conserved cysteine residues adjacent to each other (Scheme 1). Its members have a broad spectrum of activity, including attracting monocytes, eosinophils, basophils, lymphocytes, natural killer, and dendritic cells [37,38]. They are secreted mainly by neurons, immune, and glial cells [39,40,41,42].

Chemokines belonging to CC-group play important roles in the development of, among the others, autoimmune and neurodegenerative diseases, which has already been well documented [48,49,50]. Due to the huge number of members of this family, we have prepared Table 1, in which 28 chemokines and 10 of their receptors are presented. Then, in the next 10 chapters, based on data from existing studies, the role of individual receptors and their ligands after CNS and PNS damage are discussed.

**Table 1:** Changes in the mRNA and/or protein levels of chemokines from the CC family in the brain or spinal cord after central and peripheral nervous system injury: evidence from mouse/rat studies.

Chemokine	Chemokine Receptors	Central Nervous System Injury	Peripheral Nervous System Injury
**CCL1**	**CCR8**	↑ **IRBI** [51] ↑ **ICH** [52]	↑ **PSNL** [53] ↑ **CCI** [54]
**CCL2**	**CCR1, CCR2, CCR4**	↑ **TBI** [55,56,57,58] ↑ **CIBI** [59,60] ↑ **AIBI** [61] ↑ **ICH** [62] ↑ **SCI** [63] ↑ **CPSP** [64]	↑ **CCI** [48,54,65,66,67]
**CCL3**	**CCR1, CCR5**	↑**TBI** [58,68,69] ↑ **AIBI** [61]	↑ **CCI** [66,70,71,72] ↑ **PSNL** [73]
**CCL4**	**CCR1, CCR5**	↑ **TBI** [68]	↑ **CCI** [65,66,72]
**CCL5**	**CCR1, CCR3, CCR5**	↑ **TBI** [74] ↑ **IRBI** [51] ↑ **ICH** [75] ↑ **MCAO** [76]	↑ **CCI** [65,77]
**CCL6**	**CCR1**	**ND**	↑ **CCI** [65,66,78]
**CCL7**	**CCR1, CCR2, CCR3, CCR5**	↑ **TBI** [55]	↑ **CCI** [65,66,78,79,80] ↑ **SNL** [81]
**CCL8**	**CCR1, CCR2, CCR3, CCR5**	**ND**	↑ **CCI** [65]
**CCL9/10**	**CCR1**	↑ **TBI** [68] ↑ **IRBI** [51]	↑ **CCI** [65,66,82]
**CCL11**	**CCR3, CCR5**	↑ **TBI** [58] ↑ **NHI** [83]	↑ **CCI** [80]
**CCL12**	**CCR2**	↑ **TBI** [55] ↑ **ICH** [84]	↑ **CCI** [85,86]
**CCL13**	**CCR1, CCR2, CCR3, CCR5**	NOT PRESENT IN MICE/RATS	
**CCL14**	**CCR1**	NOT PRESENT IN MICE/RATS	
**CCL15**	**CCR1, CCR3**	NOT PRESENT IN MICE/RATS	
**CCL16**	**CCR1**	NOT PRESENT IN MICE/RATS	
**CCL17**	**CCR4**	↑ **TBI** [87,88] ↑ **IRBI** [51] ↑ **ICH** [89,90,91]	↑ **CCI** [92]
**CCL18**	**CCR8**	NOT PRESENT IN MICE/RATS	
**CCL19**	**CCR7**	↑ **TBI** [56]	**ND**
**CCL20**	**CCR6**	↑ **TBI** [93,94,95,96] ↑ **ICH** [97] ↑ **MCAO** [98] ↑ **SCI** [99]	**ND**
**CCL21**	**CCR7**	**ND**	↑ **CCI** [100] ↑ **SNL** [101]
**CCL22**	**CCR4**	↑ **TBI** [56,87,102] ↑ **CPSP** [103] ↑ **NHI** [104]	↑ **CCI** [92]
**CCL23**	**CCR1**	NOT PRESENT IN MICE/RATS	
**CCL24**	**CCR3**	**ND**	– **CCI** [65,80]
**CCL25**	**CCR9, CCR10**	**ND**	**ND**
**CCL26**	**CCR3, CCR10**	**ND**	– **CCI** [80]
**CCL27**	**CCR10**	**ND**	**ND**
**CCL28**	**CCR3, CCR10**	**ND**	– **CCI** [80]

**Abbreviations: *CNS injury model abbreviations:* AIBI**, acute ischemic brain injury; **CIBI**, cerebral ischemia–reperfusion brain injury model; **CPSP**, central poststroke pain; **ICH**, intracerebral hemorrhage; **IRBI**, neonatal mouse ischemia–reperfusion brain injury model; **MCAO**, middle cerebral artery occlusion; **NHI**, neonatal hypoxia-ischemia; **MCAO**, cerebral artery occlusion model; **SCI**, spinal cord injury; **TBI**, traumatic brain injury; ***PNS injury model abbreviations:* CCI**, chronic constriction injury to the sciatic nerve; **PSNL**, partial sciatic nerve ligation; **SNL**, spinal nerve ligation. Others: **↑**, enhanced chemokine level; **–**, lack of change in chemokine level; **ND**, no data available.

Generally, the CC chemokine receptors are coupled with Gαi/o. The effect of binding chemokines to their receptors inhibits AC, which negatively affects the level of intracellular cAMP and the activation of PKA [46], as a consequence. However, through the Gβγ subunit, which activates PLC-β [105], among the others, ERK1/2 is phosphorylated [106,107] and Ca^2+^ flow is increased [46,108,109]. Moreover, Gβγ affects chemokine-induced immune cell migration [110,111] through the phosphoinositide-3 kinases (PI3K), and also through the generation of phosphatidylinositol (3–5)-trisphosphate (PIP3) [112].

This review focus on documenting the changes in CC chemokine receptors and their role after damage to the nervous system in patients with various etiologies, and we have discussed whether and how blocking of CC chemokine receptors may contribute to improving therapy.

## 2. CCR1—Ligands and Pharmacological Modulation

**Scheme 2:**
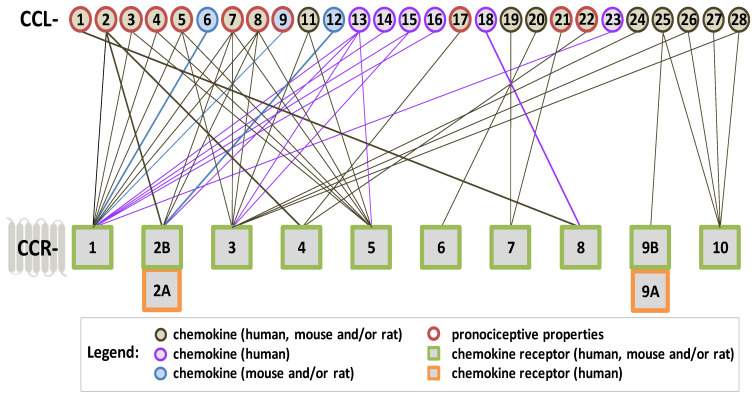
The affinity of CC-family chemokines for classic CC chemokine receptors coupled with G proteins. The algesic properties of chemokines that have been proven in behavioral studies on mice/rats are marked with a red border. Chemokines have pleiotropic properties, which we have marked in the diagram, and the lines indicate through which receptor a given chemokine acts—the purple color indicates chemokines occurring only in humans, and the blue color indicates chemokines which occur in mice/rats. Scheme created based on data from the following literature [39,40,41,53,65,73,86,100,132,139,140,141,142,143,144,145,146,147,148,149,150,151,152,153].

**CCR1** is a receptor that is involved in neuroimmunological changes after PNS and CNS injury. Experimental studies have confirmed the presence of CCR1 in many brain structures [68] and in the spinal cord [72] in mice. Notably, CCR1 is localized on many cells, including neurons [113,114], glia (astroglia and microglia) [113,114,115], and immune cells (macrophages, lymphocytes, basophils, neutrophils, and eosinophils) [116,117,118,119]. The important role of this receptor results from the fact that, in rats/mice, it is a target of many chemokines, such as **CCL2**, **CCL3**, **CCL4**, **CCL5**, **CCL6**, **CCL7**, **CCL8,** and **CCL9**, whose levels increase after brain, spinal cord and nerve injury (please see Table 1), and they are important for nociceptive transmission (Scheme 2). Notably, following NHI in rats, CCR1 expression increases in the cortex, thalamus, striatum, hippocampus, and cerebral endothelium [119]. Similarly, CCR1 upregulation in the brain and spinal cord was described 22–24 h after MCAO [76] and ICH [120] in mice. Moreover, CCR1 contributes to inflammatory processes in Alzheimer’s disease [121] and autoimmune encephalomyelitis [122]. Furthermore, the importance of CCR1 was confirmed in SNL [123] and CCI [65,66] mouse/rat models of neuropathic pain. The published data support the hypothesis that pharmacological modulation of CCR1 may represent a new strategy for effective treatment of PNS and CNS injury—however, further research, especially clinical studies, is still necessary.

### 2.1. CCR1—Endogenous Ligands

**CCL2** is a pleiotropic factor that acts as a ligand for CCR1, CCR2, and CCR4. Many studies have shown that CCL2 is one of the key immune factors upregulated in the brain after TBI [55,57,58,124,125] and in CIBI [59,60], CPSP [64], AIBI [61], ICH [62], and in the spinal cord after CCI [65,66,126,127,128] and SCI [63]. CCL2 is released by neurons, glial cells (microglia and astroglia), and immune cells (macrophages and neutrophils) [86,126,129,130,131,132,133,134]. After TBI, CCL2 expression has been shown to increase rapidly in patient cerebrospinal fluid [57] and plasma, which seems to correlate with unfavorable outcomes [135]. Experimental studies have revealed high levels of CCL2 after TBI in mouse brain structures (cortex, striatum, and thalamus), particularly in glial cells [57,124,125,136]. Importantly, several studies have shown that intrathecal administration of CCL2 induces long-lasting pain-related behavior in naive mice [86,132]. Moreover, CCL2 neutralization by antibodies or knockout by siRNA diminished hypersensitivity after CCI [86,137] and prevented glial activation [126]. Recent studies have shown that CCL2 directly contributes to the initiation and maintenance of central sensitization in CPSP model [64]; however, its pronociceptive role after brain injury has not been well studied. Moreover, CCL2 knockout mice exhibited reduced lesions in the cortex and thalamus after TBI [57]. Additionally, these animals manifested partial improvements in long-term neurological outcomes [57,138]. Furthermore, also in humans, shortly after TBI, the level of CCL2 is elevated in the CSF for up to 9 days [57]. Unfortunately, in patients after TBI, it was not possible to determine the CCL2 level in brain structures. Importantly the results from animal studies indicated that CCL2 is particularly important in the brain shortly after its injury—CCL2 upregulation in mRNA/protein level was demonstrated after 24 h not only in the cortex but also in the striatum and thalamus [55]. Moreover, research showed that CCL2 acts in the CNS not only through CCR1 but also through CCR2 and CCR4 [66,82]. However, the role of these three receptors in CCL2 effects remains to be determined. In light of the published data, the key role of CCL2 in the changes caused by PNS and CNS damage is unquestionable and suggests that it is an attractive target for new pharmacotherapies, especially in the early phase when immunological changes are initiated in the CNS.

**CCL3** acts as a ligand for CCR1 and CCR5 and is strongly upregulated in the brain after TBI [58,68,69] and AIBI [61] and in the spinal cord after CCI [66,70,71,72]. Furthermore, an increase in CCL3 was observed in model of temporal lobe epilepsy in the brain of rats [154] and lipopolysaccharide-induced brain injury [155]. Importantly, CCL3 expression was elevated in the white matter from the pericontusional area of patients shortly after post-traumatic brain contusions [156]. Notably, blocking CCL3 prevented the development of recurrent symptoms of experimental autoimmune encephalomyelitis and mononuclear cell infiltration into the CNS [157,158,159]. Studies indicated that CCL3 is produced not only by microglia and macrophages [155] but also by other cells, such as neurons, neutrophils, and lymphocytes [41,160,161,162,163]. Recently, it has been shown that the intrathecal administration of CCL3 induces pain-related behavior in naive mice [65,164], which can be reduced by the selective CCR1 antagonist BX513 [164]. Subsequent animal research has proven that CCL3 neutralization by antibodies reduces hypersensitivity evoked by CCI [72] and PSNL [73]. Recently, it has been proven that CCL3 regulates synaptic plasticity mechanisms involved in learning processes and memory in mice [165], and changes in CCL3 have also been demonstrated in rat temporal lobe epilepsy [166]. Moreover, CCL3 was found to be upregulated in the brains of Alzheimer’s patients [167,168]. Growing evidence suggests that CCL3 is upregulated in neuroinflammatory processes initiated by CNS [58,61,68,69,155] and PNS [66,70,71,72] injury. However, the exact role and regulatory mechanism of CCL3 remains unclear, and additional research should be conducted.

**CCL4** acts as a ligand of two chemokine receptors, CCR1 and CCR5, and it can be produced by neurons [169], neutrophils [170,171], microglia [41], and astroglia [172]. A strong increase in the level of CCL4 after TBI has been demonstrated in a mouse model [68], as well as in the white matter from the pericontusional area of patients after post-traumatic brain contusions [156]. In contrast, no changes in CCL4 were detected in the CCI model in the spinal cord of mice [72], likewise in streptozotocin- and chemotherapy-induced neuropathy in animals [41,173]. The results of the mentioned studies provide some evidence for the involvement of this chemokine in nociceptive processes. However, CCL4 acts as a strong chemoattractant for neutrophils [174] and is one of the main chemokines secreted by the microvascular endothelium [174]. Therefore, it may contribute to leakage through the blood–brain barrier. Moreover, the level of CCL4 is known to increase in patients affected by Alzheimer’s disease [168], osteoarthritis [175], diabetes [176], and bronchitis [177]. Although the involvement of CCL4 in nociceptive processes seems to be not crucial, contrarily, its role after CNS and PNS injury of various etiologies seems to be significant, and that is why further research is needed.

**CCL5** is the major ligand of CCR5; however, it acts also through CCR1 and CCR3. CCL5 is strongly upregulated in the brain after TBI [74], IRBI [51], MCAO [76], and ICH [75] and in the spinal cord after CCI [65,77]. CCL5 can be produced by microglial and astroglial cells [71] and is a strong chemoattractant for macrophages and lymphocytes [178,179]. However, the role of CCL5 after brain injury is generally unclear. After TBI, in the cortex of mice [180] and in the brain white matter of patients [156], the mRNA level of CCL5 increases. Moreover, the high level of CCL5 in the plasma of TBI patients correlates with severe brain injury [181,182]. However, some studies suggest that CCL5 has protective effects in mice, contributing to dendritic spine and synapse formation and improving learning and memory [183]. Similarly, the study in patients after ischemic stroke gave evidence that CCL5 has the potential to play a neuroprotective role [184]. However, published data indicated also that an increase in the expression of CCL5 in the spinal cord after PNS injury is detrimental in mice and rats [65,77,185,186]. Moreover, the pronociceptive properties of CCL5 had been proven by its intrathecal administration to naive mice [65]. Moreover, CCL5 knockout mice developed diminished pain-like behavior after PSNL [187]. In addition the neutralization of CCL5 by antibodies, there was decreased hypersensitivity development in different neuropathic pain models [77,188,189]. However, there was relatively small and short-lasting increase in CCL5 levels observed in the spinal cord after PNS and CCI, suggested that its contribution to central sensitization is less important than that of other chemokines, especially CCL2, CCL7, and CCL8. Taken together, the results obtained after PNS and CNS injury suggest that CCL5 might be an important factor for modulating inflammatory processes. However, further research is needed to understand the role of this chemokine. It is unclear whether CCL5 contributes to the development of hypersensitivity after CNS injury and whether it may have neuroprotective effects after PNS injury.

**CCL6** is a selective CCR1 ligand [190,191], and although it does not exist in humans, it is considered to have the human orthologs CCL15 and CCL23 [192]. Further investigation is needed to determine the role of CCL6 after PNS and CNS injury, and it would also be worthwhile to investigate the role of CCL15 and CCL23 in patients.

**CCL7** shares 60–71% homology with CCL2 and has been shown to trigger immune cell trafficking to sites of injury [193]. CCL7 is a pleiotropic factor that can bind to CCR1, CCR2, CCR3, and CCR5. Initially, CCL7 appeared to be involved in the development of neurodegenerative diseases, and changes in its level were associated with increased leukocyte infiltration [126,193]. Later, it was shown that CCL7 is strongly increased in the brain after TBI in mice [55]. Similarly, nerve injury strongly increases CCL7 levels in the spinal cord [65,66,78,79,80] and DRG [80], which was shown in rat and mice studies. In the CNS, CCL7 is released mostly by astroglia [194,195,196], and it is able to activate other cells [197]. However, CCL7 is also produced by activated microglia, macrophages, and neurons [197,198,199,200]. Importantly, this chemokine can evoke chemotaxis in many cells, including microglia, macrophages, and neutrophils [79,201,202], which are known to be important in neuroinflammation and nociception. It was hypothesized, based on animal studies, that the long-lasting upregulation of CCL7 in the cortex, striatum, thalamus, and hippocampus after TBI is associated with intense multidirectional neuron–glia interactions, which lead to secondary injury [55,203,204,205]. Recently, CCL7 was found to be increased after cerebral microdialysis in patients with severe TBI [206] and correlated with aging [203]. An increasing amount of research indicated that this chemokine has strong pronociceptive effects. The newest pharmacological studies provided evidence that the intrathecal injection of CCL7 evoked severe and long-term hypersensitivity in naive mice [65,86]. This finding is consistent with studies showing that CCL7 knockout mice experienced less pain than control mice [198]. Importantly, the blockade of CCL7 is more effective in relieving pain in SNL [81] and CCI [86] models than the blockade of CCL2. The above results have suggested that CCL7 is a key factor involved in the secondary changes induced by CNS and PNS injury and is a new, promising target for effective pharmacotherapy.

**CCL8** is a pleiotropic factor that can bind to CCR1, CCR2, CCR3, and CCR5 and is mostly secreted in the CNS by neurons, microglia, and macrophages [207,208]. To our knowledge, CCL8 has not been previously studied in animal models of TBI. However, in patients, it was recently found to be upregulated in the CSF after severe brain injury [206] and during neuropathy [209]. Its role after brain injury is generally unknown; however, its pronociceptive properties have already been proved in animal studies. It has been shown that intrathecal administration of CCL8 evoked a strong increase in hypersensitivity to thermal and mechanical stimuli [65]. Importantly, the latest literature data indicate that CCL8 is one of the most elevated chemokines in the spinal cord of mice after CCI, occurring from the early to late phase of neuropathic pain [65]. Based on the aforementioned data, we suggest that CCL8 may be one of the most important chemokines involved in secondary injury and nociceptive transmission. However, additional experimental and clinical studies are needed to determine its exact role in the CNS and PNS.

**CCL9** is a selective ligand of CCR1. It is not present in humans but has an ortholog—CCL23. An increase in CCL9 in the brain of mice was shown in IRBI [51] and TBI [68] models. However, among the CCR1 ligands, CCL2 [55], CCL3 [68], CCL4 [68], and CCL7 [55], they exhibited greater changes than CCL9 in the mouse model of TBI [68]. These weak and slow time-course changes of CCL9 may be related to its neuronal localization. As indicated by immunohistochemical studies, CCL9 colocalizes with NeuN [41,51] but not with IBA-1 [41] or GFAP [41,210]. The role of CCL9 still needs to be defined, and the latest research indicates that its intrathecal administration in naive mice causes tactile and thermal hypersensitivity [41,65]. Changes in the CCL9 level were also observed after CCI, and it was shown both in rats [66] and mice [65]. The mRNA level increases in the DRG and/or spinal cord at many time points; however, the protein level increases only up to seventh day [66,72]. Therefore, it was suggested that CCL9 is responsible for the initiation of neuropathic pain. Moreover, CCL9 neutralization by antibodies diminishes pain-related symptoms’ development in mouse neuropathy [41]. Based on the aforementioned data, it can be concluded that CCL9 contributes to the development of secondary damage to the CNS and to the disruption of nociceptive transmission; however, additional studies are needed to determine the exact role of CCL9 in the CNS and PNS.

The chemokines **CCL13**, **CCL14**, **CCL15**, **CCL16,** and **CCL23** are also agonists of CCR1; however, they are not present in mice or rats. To our knowledge, there are no published data concerning the roles of CCL13, CCL14, CCL15, and CCL16 after PNS or CNS injury in humans. Importantly, **CCL23** seems to be strongly involved in the inflammatory response after brain injury and may even serve as a potential biomarker for predicting patients’ prognosis after stroke and ischemia [211,212,213]. Moreover, **CCL23** is known to be upregulated in the CSF of patients with neuropathic pain [209].

Taken together, the above literature data have suggested that the levels of eight CCR1 agonists—CCL2, CCL3, CCL4, CCL5, CCL6, CCL7, CCL8, and CCL9—increase in the CNS of mice/rats after PNS/CNS injury. Therefore, these agonists appear to play a proinflammatory role, especially in secondary damage. CCL6 and CCL9 are not present in humans; therefore, CCL2/3/4/5/7/8/23-CCR1 axes can play a role after PNS/CNS injury, but clinical studies are still needed. Very strong algesic properties of CCL2, CCL3, CCL5, CCL7, CCL8, and CCL9 have already been demonstrated, particularly in animals. Importantly, it is known that all these chemokines are responsible for the development of hypersensitivity to thermal and tactile stimuli, and three of them (CCL2, CCL7, and CCL8) are also responsible for the persistence of pain.

### 2.2. CCR1—Pharmacological Modulation

Considering the strong involvement of CCR1 ligands in neuroimmune processes after brain injury of different etiologies (Table 1), we could expect numerous pharmacological studies in animal models concerning their role. However, to our knowledge, there are no data showing the effect of selective CCR1 antagonists on the development of secondary brain damage or findings from CCR1 knockout mice after CNS injury; therefore, such experiments are undoubtedly necessary. However, it has already been shown that CCR1 knockout mice exhibit reduced hypersensitivity after irritation with acetone [214] and SNL [123]. In our opinion, the above-described changes in the levels of CCR1 and its ligands, as well as the results obtained in genetically modified animals, encourage pharmacological studies in models of CNS and PNS injury. Thus far, intrathecal administration of the CCR1 antagonist J113863 has been shown to diminish mechanical/thermal hypersensitivity in mouse [65,72] and rat [66] CCI models (Table 2).

Similarly, pain relief was observed after the intrathecal administration of BX513 in SSNL rats [164] and J113863 in diabetic mice [41]. Importantly, repeated intrathecal administration of J113863 in rats reduces the activation/infiltration of microglia, macrophages, neutrophils, and lymphocytes into the spinal cord and/or DRG and in parallel with the pronociceptive interleukins IL-1β, IL-6, and IL-18 [66]. Currently, it is known that those pronociceptive factors’ spinal release from activated immune cells might attenuate opioid analgesia during neuropathy [37,48,215,216,217].

Importantly, opioids are drugs often used for the treatment of moderate-to-severe pain [218,219]; however, in neuropathic pain, their effectiveness is lower [37]. The literature clearly showed that CCR1 blockade increased morphine/buprenorphine-induced analgesia in rats exposed to CCI, probably by silencing immune factors [66]. In addition to the abovementioned interleukins, chemokines, which are CCR1 ligands, also play important roles in opioid effectiveness. Recently, it was revealed that a single administration of neutralizing antibodies against CCL2, CCL3, CCL7, and CCL9 can increase the effectiveness of opioid drugs [41,86]. Importantly, several CCR1 antagonists, such as MLN3897 for rheumatoid arthritis [220], BX471 for multiple sclerosis [220], and BAY86-5047 for endometriosis [221], are under investigation in clinical studies. These findings suggest that these antagonists can also be used in other diseases. Unfortunately, there is still a lack of clinical studies on PNS and CNS damage. In our opinion, pharmacological modulation via CCR1 may represent a novel strategy for effective therapy for neuroimmune disorders and simultaneously enhance opioid efficacy. Considering the strong proinflammatory and pronociceptive properties of CCR1 ligands, further research is undoubtedly necessary. This is especially so when taking into account how little is known about the ligands that are present in humans but not in mice or rats, e.g., CCL13, CCL14, CCL15, CCL16, and CCL23.

**Table 2:** Analgesic potential of blocking CC chemokine receptors activated by the binding of one or more chemokines after central and peripheral nervous system injury—evidence from mouse/rat studies.

Receptor(s)	Antagonist	Beneficial Effect of Antagonist after CNS Injury	Beneficial Effect of Antagonist after PNS Injury
**SINGLE ANTAGONISTS OF CC-FAMILY RECEPTORS**			
**CCR1**	**J113863**	ND	**CCI** [65,66]
	**BX513**	ND	**SSNL** [164]
**CCR2**	**RS504393**	**TBI** [222,223]; **LDH** [197]	**CCI** [48,224]; **LAMNT** [225]
	**CCX872**	**TBI** [226]	ND
	**INCB3344**	**CIBI** [227]	**CCI** [228]
	**AZ889**	ND	**CCI** [229]
**CCR3**	**SB328437**	ND	**CCI** [65,80]
	**SB 297006**	ND	**ND**
**CCR4**	**C021**	**ICH** [89,90]	**CCI** [92,230]
	**AZD-2098**	**SAH** [231]	ND
**CCR5**	**Maraviroc**	**TBI** [232,233]; **FCS** [234]; **CIBI** [235]; **ICH** [236]	**CCI** [71,85,237]
	**AZD-5672**	ND	**CCI** [72]
	**TAK-220**	**FCI** [238]	**CCI** [72]
**CCR6**	**PF-07054894**	ND	ND
**CCR7**	**-**	ND	ND
**CCR8**	**AZ084**	ND	ND
	**R243**	ND	ND
**CCR9**	**Vercirnon**	ND	ND
	**MLN3126**	ND	ND
**CCR10**	**BI-6901**	ND	ND
**DOUBLE ANTAGONISTS OF CC-FAMILY RECEPTORS**			
**CCR1/CCR3**	**UCB 35625**	ND	**CCI** [65]
**CCR2/CCR5**	**Cenicriviroc**	**TBI** [203]	**CCI** [70,85]
	**BMS-813160**	ND	ND
	**PF-04634817**	ND	ND

**Abbreviations: *CNS injury model abbreviations:* CIBI**, cerebral ischemia–reperfusion brain injury model; **FCS**, focal cortical stroke; **FCI**, focal cerebral ischemia; **ICH**, intracerebral hemorrhage; **LDH**, lumbar disc herniation; **NHI,** neonatal hypoxia-ischemia; **SAH**, subarachnoid hemorrhage; **TBI**, traumatic brain injury; ***PNS injury model abbreviations*: CCI**, chronic constriction injury to the sciatic nerve; **SSNL**, segmental spinal nerve ligation model; **IAMNT**, inferior alveolar nerve and mental nerve transection model. Others: **ND**—no data available.

## 3. CCR2—Ligands and Pharmacological Modulation

**CCR2** is also a receptor known to be strongly involved in neuroimmunological changes after PNS and CNS injury. The important role of CCR2 was demonstrated for the first time in animal models of degenerative and autoimmune diseases [239]. Subsequently, CCR2 was shown to be expressed on macrophages and leukocytes after brain injury. It is now recognized that in the CNS, CCR2 is present in microglia/macrophages, as well as in astroglia, leukocytes, and neurons in humans and mice. Although, its levels are highest in microglia/macrophages [57,132,240,241,242,243,244]. CCR2 is preferentially bound by CCL2 [245]; however, several other chemokines, CCL7, CCL8, CCL12, and CCL13 (please see Table 1), can also bind to this receptor [37,239,245,246]. Importantly, CCR2 activation promotes angiogenesis [247] and fracture healing [248] and decreases amyloid plaque formation in Alzheimer’s disease models through macrophage-mediated phagocytosis [249,250]. On the other hand, CCR2 plays an unfavorable role and has been documented in many animal models, e.g., TBI [55,56,57,58,251], CIBI [59,60,252], AIBI [61], ICH [62], SCI [63], CPSP [64], EAE [253], diabetic retinopathy [254], cancer [255], arthritis [256], and neuropathy [48,70,257]. After brain injury, CCR2 knockout mice exhibit reduced macrophage infiltration, improved hippocampus-dependent cognitive outcomes, and preserved hippocampal neurons viability [57,226,258]. Similarly, after CCI, CCR2 knockout mice develop diminished hypersensitivity, which is associated with decreased infiltration of CCR2-positive macrophages [259]. The recruitment and activation of those cells in the CNS are known to significantly contribute to the development of both inflammation and neuropathic pain. Therefore, CCR2 receptor blockade may provide a new therapeutic method for the treatment of CNS and PNS disorders.

Importantly, the CCR2 gene encodes two isoforms, CCR2A and CCR2B, which feature different amino acid sequences in their C-terminal intracellular loops due to alternative splicing [260,261]. Most biochemical and behavioral studies on CCR2-related responses have focused on CCR2B, with few reports focused on CCR2A. A study by Bartoli et al. showed that CCR2 isoforms are cell-specific in humans [262]. CCR2A is the major isoform expressed by mononuclear cells, and CCR2B is expressed by satellite cells [262]. CCR2B is the most abundant form; however, both CCR2 isoforms are expressed on different immune (monocytes, lymphocytes, and dendritic cells) [239] and endothelial [263,264,265] cells. Importantly, CCR2A, but not CCR2B, is expressed in solid cancer-derived cells, indicating that CCR2A may play an essential role in tumor progression. The results revealed that the C-terminal region of CCR2A is unfavorable for GRK phosphorylation, which is necessary for β-arrestin interaction and subsequent receptor internalization [266]. Furthermore, CCR2A is expressed in vessel walls and by some mononuclear cells, especially in cells involved in partial invasion in polymyositis and inclusion body myositis [262]. According to the Ensembl genome analysis database, the splicing process used to produce CCR2A may be unique in some primate species, and both of these transcripts have been identified in humans [266]. Since the rodent CCR2 gene does not undergo alternative splicing and thus does not generate CCR2A, mice/rats are not good models for exploring the tissue expression and biological functions of CCR2A [266]. Therefore, the presence and role of CCR2A and CCR2B in the CNS/PNS in humans need to be studied.

### 3.1. CCR2—Endogenous Ligands

As described above, many studies have shown that chemokine CCL2, CCL7, and CCL8 (which are known to bind to several receptors; Scheme 2) levels are increased after PNS/CNS injury [37,245]; therefore, they appear to play a role in neuroimmune disorders, especially secondary damage and nociceptive transmission. The newest data indicate that CCL2, CCL7, and CCL8 are multidirectional factors responsible for communication between neurons, microglia, and astroglia; thus, they are essential for the functioning of the nervous system. However, the possible involvement of CCL13 in neuroimmune processes remains to be studied since this chemokine does not exist in mice/rats, making its role more difficult to clarify. We know relatively little about the role of CCL12—the selective ligand of CCR2. However, its upregulation was already described in various animal models, such as in TBI [55,68,203], ICH [84], CCI [85,86,267], and osteoarthritis [268,269]. Importantly, in contrast to CCL2, CCL7, and CCL8, CCL12 is devoid of pronociceptive properties [65,86]; however, after CNS injury, it exerts potent proinflammatory effects by inducing leukocyte, macrophage, and T-cell chemotaxis [84,270,271]. The intrathecal injection of CCL12-neutralizing antibodies is neuroprotective after brain injury [84]. Interestingly, the changes in CCL12 in the hippocampus seem to be age-dependent [203]. Therefore, the blockade of CCL12 may constitute a new therapeutic approach for the treatment of CNS damage, e.g., occurring along with the aging process, but further in-depth research is needed.

### 3.2. CCR2—Pharmacological Modulation

Increasing amounts of published data indicate that the pharmacological modulation of CCR2 may be beneficial in the treatment of neuroimmune disorders. Results from CCR2 knockout mice have shown both significantly improved cognitive function after TBI [258] and reduced hypersensitivity after CCI [259], which encourages pharmacological studies. Literature data indicate that the selective CCR2 antagonist RS504393 reduces apoptosis and improves performance in the Morris water maze after TBI, suggesting again that this receptor has deleterious effects on neuronal survival and learning [222,223]. Subsequent research has shown that after stroke, monocyte numbers increase significantly in both the blood and brain (even up to 10-fold), and the new CCR2 antagonist INCB3344 selectively prevents these changes. Additionally, INCB3344 administration limits brain damage and improves functional deficits, which are associated with promoting M2 macrophage polarization [227,228]. Importantly, recent studies have shown that blocking CCR2 with the new antagonist CCX872 immediately after TBI is highly beneficial because it reduces the type I IFN response [272] and blocks the influx of CCR2^+^ macrophages [226] in acutely injured brain tissue in mice. Notably, this antagonist is currently used in clinical trials for the treatment of patients diagnosed with pancreatic cancer [273], and if it passes clinical trials successfully, it could be used to treat CNS damage in the future. Importantly, extensive literature data clearly suggest that CCR2 also plays an extremely important role after PNS injury. It has been shown that repeated intrathecal injection of RS504393 decreases CCI-evoked hypersensitivity in mice and rats [48,85]. Moreover, RS504393 administration weakens microglial cell activation [224] and prevents the upregulation of interleukins (IL-1β, IL-18, and IL-6) in the spinal cord [48] and chemokines (CCL2, CCL3, CCL4, CCL5, CCL7, and CCL11) in the DRG in rats [85]. Moreover, the intrathecal administration of other CCR2 antagonists, INCB3344 [228] and AZ889 [229], also attenuated neuropathic pain symptoms in rats. Similar analgesic effects of intracisternal injections of RS504393 were described after inferior alveolar nerve transection [225], mental nerve transection [225], and lumbar disc herniation [274]. However, a single intraperitoneal injection of RS504393 in mice did not diminish already well-established neuropathic pain-related behavior, which may indicate poor passage of this compound through the BBB [85]. The effectiveness of CCX872 would undoubtedly be worth checking due to its beneficial effect on brain damage, but for now, there are no such data in the literature concerning its efficacy after nerve damage. This approach would also be valuable because it has been shown that repeated intrathecal injection of RS504393 enhances the analgesic effects of opioid drugs (morphine and buprenorphine) [48]. Further research is needed to determine why this is possible, but it is already known that excessive production of interleukins, such as IL-1β and IL-18, might be key for reducing opioid analgesia in neuropathy [215,216,275]. Several studies have indicated that opioid signaling is connected not only to interleukins but also to chemokines [37,246]. CCR2 is preferentially bound by CCL2 [245], but repeated administration of morphine increases the level of CCL2 in the spinal cord; however, its neutralization by an antibody diminishes the development of morphine tolerance [276]. Furthermore, an agonist of the μ opioid receptor (DAMGO) was shown to enhance the expression of CCL2 in human peripheral blood mononuclear cells [277]. CCL2 is known to interfere with the analgesic effects induced by opioids through heterologous desensitization between CCR2 and opioid receptors [278]. These effects correspond well with the reductions in CCI-induced elevation of CCL2, IL-1β, IL-18, and IL-6 [48,224]. Therefore, intrathecally administered RS504393 seems to inhibit heterologous desensitization between opioid and chemokine receptors by preventing the CCI-induced increase in the expression of these anti-opioid factors, although this still needs to be proven. In summary, the available data suggest that the pharmacological blockade of CCR2 beneficially influences immunological changes and, therefore, it may constitute a new strategy for effective therapy in patients suffering from CNS and PNS damage. However, additional studies are needed, possibly with the use of new pharmacological tools.

In summary, the abovementioned literature data suggest that in mice/rats treated with CCR2 agonists, CCL2, CCL7, CCL8, and CCL12 levels are increased after PNS/CNS injury; therefore, these cytokines appear to play proinflammatory roles, especially in secondary CNS damage. However, in humans, CCL12 is not present. After injury to PNS/CNS in patients, the CCL2/7/8-CCR2 axis likely plays proinflammatory and pronociceptive roles.

## 4. CCR3—Ligands and Pharmacological Modulation

**CCR3** seems to be involved in neuroimmunological changes after PNS and CNS injury [65,83,279,280] because it is a target of numerous chemokines, including CCL5, CCL7, CCL8, CCL11, CCL13, CCL15, CCL24, CCL26, and CCL28 (please see Table 1). Moreover, in the nervous system, CCR3 is expressed in neuronal, glial (microglia, astroglia, and satellite cells), and immune (basophils, eosinophils, neutrophils, and lymphocytes) cells [80,281,282,283,284,285,286,287,288]. To date, data in the literature indicate the important role of CCR3 in disorders such as inflammation, asthma, allergies, and cancer [287,289,290,291,292,293] and, recently, in neuropathy [65,80,294]. In 2013, for the first time, after ischemic injury in a mouse model was shown, the expression of CCR3 was induced in neurons around the peri-infarct areas [184]. In vitro studies have shown that CCR3 knockout and blockade protect neurons from oxygen/glucose deprivation-induced cytotoxicity in primary cortical cultures [282]. Moreover, after ischemic injury, CCR3 knockout mice exhibited a decrease in infarct volume in the brain [282], which provided the first evidence that this receptor is involved in neuronal death. All these findings confirm the need for additional research on the role of CCR3 in the nervous system to determine whether blocking this receptor may contribute to more effective treatment of PNS or CNS damage.

### 4.1. CCR3—Endogenous Ligands

The proinflammatory properties of CCL5, CCL7, and CCL8 are already well documented due to their pleiotropic effects and occurrence in various mammalian species, while the roles of CCL13 and CCL15, which are absent in mice/rats, are poorly understood. Importantly, little is known about the remaining CCR3 ligands (CCL11, CCL24, CCL26, and CCL28), although they are present in rodents and humans. Notably, three CCR3 agonists (CCL5, CCL7, and CCL8, which are described in detail above) are important pronociceptive factors (Scheme 2).

**CCL11** acts via CCR3, CCR5, and CXCR3. This chemokine is secreted in the CNS by microglia and astroglia [114,295] and is a strong chemoattractant for eosinophils, basophils, neutrophils, and macrophages [296,297]. Recently, it was shown that CCL11 is upregulated in TBI [58] and NHI [83] animal models; however, its role needs to be clarified. Similarly, in the CCI model, the level of mRNA increases in the spinal cord in rats [80] but not in mice [65]. However, CCL11 protein levels are increased in the DRG but not in the spinal cord after CCI in rats [80]. Importantly, it was recently shown that CCL11 expression increases in the CSF of patients with neuropathic pain, suggesting its potential role in nociception [209]. In particular, in patients with fibromyalgia [298] and osteoarthrosis [299], CCL11 levels are enhanced. Recently, clinical trials of the anti-CCL11 monoclonal antibody (bertilimumab) have been underway, and the preliminary results indicate that this antibody has some beneficial effects on the treatment of severe allergic disorders, skin diseases, and inflammatory bowel disease [300,301]. Considering the promising results of pharmacotherapy with bertilimumab, further research is undoubtedly necessary in patients after PNS/CNS damage.

To date, the other three CCR3 ligands, **CCL24**, **CCL26**, and **CCL28**, have been poorly investigated in the CNS and preliminary results vary among species. It is known that in rats after CCI, CCL28 mRNA is undetectable in the spinal cord [80]. However, in mice, the mRNA levels of CCL24 [65] and CCL28 [65] are detectable; however, they remain unchanged after injury. Moreover, in rats, CCL26 expression is slightly spinally elevated after CCI, but it remains unchanged in mice [65,80]. That is why the role of these chemokines after PNS/CNS injury still needs to be investigated.

### 4.2. CCR3—Pharmacological Modulation

Studies on the impact of pharmacological modulation of CCR3 on the development of neuroimmune changes in animal models of CNS damage should be conducted soon in light of the published data suggesting the role of its ligands (CCL5, CCL7, CCL8, and CCL11). Additionally, little is known about the extent to which modulation of this receptor influences neuroimmunological changes after PNS damage. In 2021, the first paper proving the key role of CCR3 in nociceptive transmission was published [80]. These studies demonstrated for the first time that blocking CCR3 through repeated intrathecal injections of SB328437 attenuates the development of hypersensitivity in a rat model of CCI [80]. In other studies, the authors showed that SB328437 administered in this way prevents the CCI-induced activation of lymphocytes, neutrophils, and satellite cells in the spinal cord and DRG and consequently results in a reduction in the level of proinflammatory cytokines (IL-6, CCL7, and CCL11), which are known to have anti-opioid properties [80]. Further studies in mice and rats have shown that even single administrations of SB328437 have analgesic effects [65,80] and, importantly, increase the effectiveness of morphine and buprenorphine in a neuropathic pain model [80]. Further research is needed to determine whether this beneficial effect on opioid efficacy is caused by the silencing of immunological factors or whether heterologous desensitization of CCR3-MOR is possible (as already been demonstrated in the case of CCR5-MOR [302,303,304]). Additionally, SB328437 has been shown to reduce hypersensitivity in the mice pIONT neuropathy model [294]. Moreover, it was shown that CCR3 plays an important role in the pathogenesis of osteoarthritis [280], allergic asthma [305], and cancer [306] in patients. In summary, the available literature data support the theory that the pharmacological blockade of CCR3 may be a new strategy for effective pharmacotherapy of PNS and CNS disorders; however, further research using newly synthesized antagonists is undoubtedly necessary.

Taken together, the above literature data suggest that among CCR3 agonists, the levels of CCL5, CCL7, CCL8, and CCL11 increase after PNS/CNS damage. Therefore, the CCL5/7/8/11-CCR3 axis appears to play a proinflammatory and pronociceptive role, especially in secondary CNS damage; however, further studies are needed.

## 5. CCR4—Ligands and Pharmacological Modulation

**CCR4** is a target of two selective agonists, CCL17 and CCL22, and one nonselective agonist, described above, CCL2 (please see Table 1) [82,108,307]. The receptor is expressed on numerous immune cells (including lymphocytes, platelets, natural killers, and macrophages) [108,307,308] and on neurons [166], microglia [286], and astroglia [108,286]. Recently, the upregulation of CCR4 expression was described in peripheral blood monocytes stimulated with serum collected from TBI patients [309]. Moreover, CCR4 expressed on immune cells was shown to play a role in cell migration and polarization [310,311,312,313]. The role of this receptor in the CNS was suggested in many animal studies after CNS injury (in a mouse MCAO model [312]), after PNS injury (in mouse/rat CCI models [92,230]), and in diabetes in monkeys [314] and mice [315]. Furthermore, CCR4 has been shown to play an important role in the pathogenesis of many diseases in patients, such as TBI [309], asthma [316,317], dermatitis [318], and cancer [319]. The presence of CCR4 in the CNS [320,321] and PNS [186] suggests that CCR4 plays a role after injury; however, further studies are needed.

### 5.1. CCR4—Endogenous Ligands

The proinflammatory properties of CCL2 are quite well documented, as described above. Importantly, due to the pleiotropic properties of this chemokine, it is one of the best studied. Recent pharmacological studies have proven that intrathecally administered CCL2 evoked pain-related behavior in naive mice more effectively through CCR4 than through CCR2 [230]. However, both the CCL2/CCR2 [48,224,245] and CCL2/CCR4 [230] axes seem to be important in the nervous system, and further in-depth research is needed. Moreover, there is still insufficient information in the current literature regarding the role of CCR4 and its selective ligands (CCL17 and CCL22) after CNS and PNS injury.

**CCL17** is a selective ligand of CCR4 and is produced mainly by thymic cells and is also secreted by lymphocytes, macrophages, microglia, dendritic cells, and neurons [82,322,323]. Recently, CCL17 was shown to be upregulated in brain structures in several animal models of TBI [87,88], IRBI [51], and ICH [89,90,91]. Moreover, although CCL17 levels in the spinal cord remain unchanged after nerve damage [66,230], its levels in the rat DRGs significantly increased [92]. Furthermore, pharmacological studies have confirmed that intrathecally administered CCL17 evoked strong pain-related behavior in naive mice through CCR4 [230]. Targeting this chemokine has not been extensively tested in clinics, but recent reports have shown that CCL17 is important for patients suffering from ICH [91], diabetic retinopathy [324], and fibromyalgia [298]. Therefore, the role of CCL17 after PNS/CNS injury urgently needs to be studied.

**CCL22** is also a selective ligand of CCR4 that can be produced by immune cells, mainly macrophages, and is able to mediate monocyte, lymphocyte, and natural killer chemotaxis [82,325,326,327]. Both CCL17 and CCL22 bind to CCR4, and these chemokines have 37% identity at the amino acid sequence level [328]. However, CCL17 and CCL22 are not equivalent in receptor binding, and CCL22 acts as the dominant cross-desensitizing ligand toward CCR4 [329]. The role of CCL22 is still unclear, but it seems to be dualistic since chemokines exhibit both pro- and anti-inflammatory effects depending on the circumstances [330]. Downregulation of CCL22 was shown to be correlated with the attenuation of neurological changes after injury in rats [56]; however, others have proposed that CCL22 is an anti-inflammatory factor responsible for M2 microglial polarization [102,330]. Our unpublished results demonstrated time-dependent changes in the mRNA and protein expression of CCL22 within the cortex, striatum, and thalamus after TBI in mice, and the data are similar to those of other studies [56,87]. Moreover, it was shown that the level of CCL22 is increased in the cortex after concussion in rats [102], ischemic stroke in mice [103], and also in patients with fibromyalgia [298]. Moreover, CCL22 was shown to play an important role in a mouse neonatal hypoxic-ischemic brain injury model [104]. These data strongly suggest that CCL22 plays a role in the pathology of brain injury. The situation seems to be different after nerve injury, since the spinal level of CCL22 remains unchanged, unlike the level of CCL2, which was highly increased [230]. However, after CCI, the level of CCL22 is strongly increased in rats, similar to CCL2 [230]. In summary, the role of the CCL22/CCR4 axis at the PNS and CNS levels still needs to be clarified, but its involvement in pathological changes is unquestionable.

### 5.2. CCR4—Pharmacological Modulation

Recently, neutralizing molecules for CCL17 and CCL22 (GPN279 and GPN136) were created, and they have been shown to have anti-inflammatory effects in a mouse model of asthma [331]. Moreover, neutralization of CCL17 using antibodies has been shown to reduce the proliferation of HeLa and SiHa tumor cells [332], and CCL22 delays the development of experimental autoimmune encephalomyelitis [333]. Due to the favorable properties of neutralizing molecules CCL2 (described in Section 2.1), CCL17, and CCL22, these chemokines appear to be particularly important targets for future therapies.

Currently, four groups of CCR4 antagonists, distinguished based on their chemical properties, are available for experimental research: aryl sulfonamides, substituted heterocyclic amines, thiazolidinones, and lactams [334,335]. However, of these antagonists, only indazole arylsulfonamide (GSK 2239633) was tested in phase I clinical trials, and further studies were not performed [108,336]. Recently, a CCR4 antagonist called C021 was experimentally tested, and it is known that it is able to inhibit the chemotaxis of immune cells in mouse and human in vitro studies [337,338]. C021 has also been shown to reduce microglial activation in animal models of hepatic encephalopathy [339] and neuropathic pain [92,230]. Importantly, it was shown that repeated intrathecal and intraperitoneal injections of C021 diminish pain and, in parallel, spinal levels of macrophage/microglia activation [92,230] and, consequently, the levels of the proinflammatory cytokines IL-1β and IL-18 in rats [92]. Moreover, in CCI-exposed rats and mice, C021 administration improved morphine and buprenorphine analgesia [92,230] and reduced the development of morphine tolerance [230]. Finally, we would like to emphasize that clinical trials of mogamulizumab (a humanized CCR4 antibody) are currently ongoing in Japan. Mogamulizumab is being tested for the treatment of T-cell lymphomas and leukemia [340,341] and advanced solid tumors [341]. Moreover, the use of mogamulizumab has also been tested in allergic diseases, such as asthma and atopic dermatitis [307]. However, this drug remains in the early stages of clinical studies, and further research is needed. In summary, published research has indicated that targeting CCR4 and its ligands is a promising strategy for treating nervous system damage with various etiologies in animal models.

In summary, the abovementioned literature data suggest that in humans and also in rodents like mice/rats, the expression of all CCR4 agonists is increased after PNS/CNS injury. Therefore, the CCL2/17/22-CCR4 axis appears to play a proinflammatory and pronociceptive role, especially in secondary CNS damage. However, further clinical studies are needed.

## 6. CCR5—Ligands and Pharmacological Modulation

**CCR5** is known to be strongly involved in neuroimmunological changes after PNS and CNS injury, which is not surprising since it is a target of several chemokines, **CCL3**, **CCL4**, **CCL5**, **CCL7**, **CCL8**, **CCL11**, and **CCL13** (please see Table 1). CCR5 expression is well documented in a variety of immune cells (macrophages, lymphocytes, and granulocytes) and glia (microglia and astroglia) but was recently found on neuronal cells [342,343]. Importantly, stroke induces CCR5 expression in neurons, and this expression persists throughout recovery. The downregulation of CCR5 in microglia at similar time points when CCR5 expression is increased in neurons reflects complex cell-specific changes in CCR5 signaling after stroke. Recently, CCR5 was shown to be involved in learning, memory, and plasticity processes in hippocampal and cortical circuits [344]. In a TBI model, CCR5 knockout mice exhibited reduced learning deficits and improved cognitive function [345]. Importantly, after stroke, patients who were carriers of a naturally occurring loss-of-function mutation in CCR5 (CCR5-Δ32) exhibited improvements in cognitive and neurological disorders [345]. Moreover, CCR5 knockout mice developed hypersensitivity to a lesser extent than control mice [304]. Therefore, the CCR5 receptor appears to be a therapeutic target for the treatment of pain-related diseases, including neuropathy [237,346,347,348]. In summary, CCR5 is proposed to be a pharmacological target for regeneration and/or pain relief after CNS and PNS injury [237,345,346,347,348].

### 6.1. CCR5—Endogenous Ligands

Notably, CCR5 agonists, namely, CCL3, CCL4, CCL5, CCL7, CCL8, and CCL11, exhibit pleiotropic properties and have potent and well-documented proinflammatory and/or pronociceptive properties in the CNS and/or PNS. However, there is a lack of information about the role of CCL13, a chemokine found in humans but not in mice/rats, after PNS or CNS damage. Due to the well-documented role of the abovementioned six CCR5 ligands, this receptor seems to be a particularly valuable drug target.

### 6.2. CCR5—Pharmacological Modulation

Literature data clearly indicate that CCR5 plays an important role in recovery after brain injury of different etiologies (please see Table 1) [75,232,233,234,235,236,238,345]. Poststroke neuronal knockdown of CCR5 in the motor cortex led to the early recovery of motor control in mice [345]. It was proven that this recovery is associated with the preservation of dendritic spines and novel patterns of cortical projections to the contralateral premotor cortex [345]. Importantly, it has been proved that stroke induces strong CCR5 expression in neurons. This process is accompanied by a simultaneous reduction in the level of CCR5 in microglia, which indicates how complex the neuroimmunological changes after CNS damage are [345]. Notably, the administration of maraviroc, a CCR5 antagonist clinically used for HIV infection treatment, has been shown to produce similar effects on motor recovery after stroke and cognitive decline after TBI in mice [345]. The beneficial effects of maraviroc have also been shown by others in various animal models, such as TBI [192,193], stroke [194], and CIBI [195] models. Additionally, TAK-220, a potent and selective antagonist of CCR5, was shown to be protective against focal cerebral ischemia in mice [197]. Importantly, it has recently been shown that after stroke, patients who carry a naturally occurring loss-of-function mutation in CCR5 (CCR5-Δ32) show better improvement in neurological and cognitive disorders [345]. Therefore, CCR5 can be considered an important pharmacological target after CNS injury. Furthermore, CCR5 is the first human chemokine receptor known to play a role in improving recovery from stroke and ICH [75].

Importantly, after PNS injury, CCR5 is upregulated in the spinal cord, which was also shown in a rat CCI model [71]. Therefore, the blockade of CCR5 after PNS injury has also beneficial effects. In a mouse CCI model, the administration of the CCR5 antagonists maraviroc [85,237], AZD-5672 [72], and TAK-220 [72] was shown to diminish hypersensitivity to tactile and thermal stimuli. Moreover, in rats, intrathecal administration of maraviroc was shown to diminish pain symptoms in a CCI model and, in parallel, spinal microglia/astroglia activation, which are important for pain development. These activated cells are also responsible for the release of many anti-opioid immune factors, such as IL-1β [216], IL-18 [215], CCL3 [41,86,349], and CCL5 [77,188,189]. In addition, morphine administration enhances the level of CCR5 in lymphocytes [348]. Therefore, blocking CCR5 with maraviroc [71,85], AZD-5672 [72], and TAK-220 [72] not only diminished pain but also enhanced opioid effectiveness in mouse and rat neuropathic pain models. Moreover, the latest research indicates that heterologous desensitization of opioid and chemokine receptors may occur at the CNS level, as it has been shown that CCR5 and mu and kappa opioid receptors are present on glial and neuronal cells [187,348,350,351]. Considering the abovementioned literature, we believe that CCR5 is a good target for drug development in the treatment of CNS and PNS injury and that maraviroc seems to be a drug that should be subject to clinical trials. In particular, maraviroc has received approval from the Food and Drug Administration and is used as a treatment for patients infected with human immunodeficiency virus R5 type 1 [352,353]. The clinical use of maraviroc may weaken the development of secondary changes after damage to the CNS and PNS, reduce the development of pain hypersensitivity, and enhance the analgesic effects of opioid drugs in patients treated for neuropathy.

In summary, the abovementioned literature data suggest that in mice/rats and also in humans, the expression of the CCR5 agonists CCL3, CCL4, CCL5, CCL7, CCL8, and CCL11 increases after PNS/CNS injury. Therefore, the CCL3/4/5/7/8/11-CCR5 axis appears to play a proinflammatory and pronociceptive role, especially in secondary CNS damage; however, additional clinical studies are needed.

## 7. CCR6—Ligands and Pharmacological Modulation

**CCR6** and CCL20 are unique in that they interact only with each other (please see Table 1). The receptor is expressed on immature dendritic cells, B and T cells, and neutrophils [354]. There are limited data in the literature about the role of the CCL20/CCR6 axis after PNS and CNS injury; therefore, further studies are necessary.

### 7.1. CCR6—Endogenous Ligands

**CCL20** is expressed by endothelial and epithelial cells, monocytes, and Th17 lymphocytes [355] and attracts immature dendritic cells and T and B cells [354]. In brain tissue, CCL20 is localized mainly to neurons and microglia, while little CCL20 was found in astroglia [356]. Studies have shown that CCL20 expression was increased after TBI [93,94,95], ICH [97], cerebral ischemia [98], and spinal cord injury [99] in rodents. CCL20 levels have also been shown to be elevated in human plasma after severe TBI [181]. Available data showed that the binding of CCL20 to CCR6-positive T cells is crucial for their infiltration into the CNS [357]. Recently, after injury or inflammation, activated astroglia that form the blood–brain barrier produce CCL20, which makes it easier for CCR6-positive cells to infiltrate the CNS [358]. Moreover, activation of the CCL20-CCR6 axis seems to be important in the development of dry eye disease, which affects approximately 37% of TBI patients [359]. Interestingly, a recent study reported that CCL20 expression is upregulated after ICH in mice [356] and rats [360] and that chemokine was suggested as a potential target for pharmacological intervention. The algesic properties of CCL20 have not yet been documented. However, CCL20 levels were shown to increase after breast tumor surgery in patients who developed persistent postoperative neuropathic pain [361]. Similarly, an increase in the plasma CCL20 concentration was shown to be associated with hypersensitivity in patients after nerve damage [362]. However, further investigation is needed to determine the underlying molecular mechanisms through which CCL20 influences neuroimmunological changes.

### 7.2. CCR6—Pharmacological Modulation

A selective CCR6 antagonist (PF-07054894) can be used in experiments, but to our knowledge, such studies have not yet been carried out after damage to the CNS and PNS. However, treatment with shCCL20-CCR6 nanodendriplexes improved pathology in mice after TBI [96]. Importantly, CCR6 knockout mice exhibited significant protection against retinal damage, which is often induced by TBI [93]. After spinal cord injury, CCL20-neutralizing antibodies helped to restore motor functions and inhibited upregulation of TNF-α, IL-1β, and IL-6 [356]. The expression of CCL20 and CCR6 was found to be upregulated in ischemic brain injury, while a CCL20-neutralizing antibody reduced the volume of cerebral infarction in mice [356]. However, the contributions of CCL20 to the pathology of early brain injury after SAH and its underlying mechanisms are still unclear. In conclusion, further research is necessary to elucidate the role of the CCL20-CCR6 axis after CNS and PNS injury.

In summary, the abovementioned literature data suggest that in mice, rats, and humans, the levels of the CCR6 agonist CCL20 are increased after PNS/CNS injury. Therefore, the CCL20-CCR6 axis seems to play a proinflammatory role in patients after PNS/CNS injury. However, additional advanced studies on this issue are still needed.

## 8. CCR7—Ligands and Pharmacological Modulation

There are two known ligands for **CCR7**, CCL19 and CCL21 (please see Table 1) [363]. In 2010, it was shown that CCR7 is upregulated in astroglia activated by LPS, which suggested that this receptor is important during CNS inflammation [364]. CCR7 levels are diminished in hippocampal neurons during pilocarpine-induced status epilepticus in mice [365]. CCR7 is expressed in the nervous system on several immune cells, including T and B cells, lymphocytes, and dendritic cells [363]. The role of CCR7 is not sufficiently understood, but CCR7 stimulation causes dendritic cell maturation [363]; moreover, CCR7 knockout mice more frequently develop autoimmune disorders. In the light of the available literature, the roles of the CCL19/CCR7 and CCL21/CCR7 axes in CNS and PNS damage are unclear, and further in-depth research is needed.

### 8.1. CCR7—Endogenous Ligands

**CCL19** is known as a strong chemoattractant for leukocytes and B/T cells in the brain after injury [56]. Moreover, the level of CCL19 is increased in the cerebrospinal fluid of patients with neuropathic pain [209,366]. Additionally, it was shown that this chemokine plays a key role in rat orofacial pain development [367]. In light of the scarce literature, the role of CCL19 in the neuroimmune changes caused by damage to the PNS and CNS should be determined.

**CCL21** is expressed exclusively in damaged neurons of the CNS, and CCL21 rapidly induces inflammation because it strongly activates microglia [101,368]. Importantly, CCL21 exerts proinflammatory effects through two receptors: CCR7 and CXCR3 [369]. It has been shown that in a spinal cord injury model, mice with low CCL21 gene expression exhibit weakened classic microglial activation, which is associated with the proinflammatory phase [27]. Importantly, in the CCI model, an increase in the level of CCL21 in the spinal cord was also demonstrated, and intrathecal administration of CCL21-neutralizing antibodies inhibited the development of hypersensitivity to thermal and tactile stimuli [100]. Moreover, the presence of CCL21 in the serum of patients is an independent risk factor for cognitive impairment after spinal cord injury [370]. Therefore, CCL21 may be an important target for pharmacological intervention in neuroinflammation after spinal cord injury. Undoubtedly, CCL21 plays an important role in hypersensitivity development [368,371]. However, the CCL21/CXCR3 axis is likely more important than the CCL21/CCR7 axis for neuropathic pain [372] and needs to be studied further.

### 8.2. CCR7—Pharmacological Modulation

CCR7 consists of 353 amino acids [373], and recently, it was shown that its crystal structure contains an allosterically bound antagonist termed Cmp2105 [374,375]. Therefore, additional studies of CCR7 are necessary to determine the therapeutic potential of its intracellular allosteric pocket. Currently, some known substances are weak inhibitors of CCR7 [376]. N-truncated CCL19 was shown to bind to but not activate CCR7 by binding to the extracellularly exposed part of the receptor and preventing the binding of CCL19 and CCL21 [376,377,378,379]. Moreover, cosalane, a cholesterol derivative designed as a therapeutic for human immunodeficiency virus, can inhibit CCR7 in response to both CCL19 and CCL21 agonists in humans and mice [376]. To our knowledge, there are no published data showing the effects of CCR7 blockade or deficiency after damage to the nervous system. To date, the published data revealed that the deletion of CCR7 is not lethal in mice. Moreover, CCR7−/− mice exhibit impaired early production of specific IgG isotype antibodies in response to the antigen, failure to initiate fast primary B- and T-cell responses, and impaired homing of immune cells [380,381,382]. Interestingly, mice with CCR7 deletion exhibit an enhanced immune response to a variety of stimuli, including contact hypersensitivity reactions. B cells from these mutant strains are partially capable of migrating to the lymph nodes and splenic white pulp, which only proves that CCR7 is not the only regulator of lymphocyte mobilization [380,382]. Undoubtedly, research is necessary to determine the impact of CCR7 modulation on nervous system function after injury.

In summary, the above literature data suggest that in mice/rats and also in humans, CCR7 agonists, i.e., CCL19 and CCL21, may be important after PNS/CNS injuries. However, confirmation of these findings in further basic and clinical studies is needed. For nociceptive transmission, the CCL21/CXCR3 axis is likely more important than the CCL21/CCR7 axis is.

## 9. CCR8—Ligands and Pharmacological Modulation

Although **CCR8** was discovered relatively long ago, in 1997, its role in the CNS and PNS still remains poorly understood [383]. CCR8 is known to have two selective ligands, CCL1, which was described in 1989 [384], and CCL18, which was discovered in 2013 [385] (please see Table 1). However, due to false reports of the absence of CCR8 in human peripheral immune cells and due to the lack of phenotypic abnormalities in CCR8 knockout mice, intensive research on this receptor was not conducted after its discovery [386]. Nevertheless, it was recently shown that this receptor is present on various types of lymphocytes, mainly in the skin but also in peripheral blood [386]. CCR8 is already known to play an important role in immune-mediated disorders, both in patients with atopic dermatitis [387] and in mouse models of allergic enteritis [388] and diabetes [153,389]. Importantly, recent immunofluorescence staining of the CNS demonstrated that CCR8 is highly expressed in neurons [153], suggesting that the initial assumptions about its nonessential role were most likely incorrect and that further studies are needed to determine its participation in changes after damage to the CNS and PNS. Importantly, the first published data suggested that in the mouse PSNL model, an increase in the CCR8 levels in the spinal cord is observed in both neuronal and glial cells [53], which indicates a possible important role for this receptor in the CNS.

### 9.1. CCR8—Endogenous Ligands

**CCL1** was initially thought to be a chemokine produced primarily by lymphocytes, but recently, it was shown that it can also be secreted by many other cells, including neurons, monocytes, mast cells, and epithelial/endothelial cells [153,390]. The currently available literature indicates that CCL1 most likely plays an important role in the CNS and PNS as a mediator of neuron–glia interactions, which is why we believe that it may contribute to the progression of secondary disorders of the nervous system, including the development of neuropathic pain [391]. Importantly, an increase in CCL1 levels in mice was observed in the brain after ICH [52] and CIBI [51] and also in the spinal cord after CCI and PSNL. Importantly, the CCL1 changes occur after nerve injury in parallel with the development of neuropathic pain [53,153,392]. Notably, subsequent studies have indicated that pain symptoms are reduced after the intrathecal injection of a CCL1-neutralizing antibody [53,153] and that hypersensitivity developed to a lesser extent in CCR8 knockout mice [53]. Other studies have provided direct evidence for the pronociceptive effects of CCL1—as recombinant CCL1 administered intrathecally to naive mice induces thermal and tactile hypersensitivity [53,153] and parallel activation of spinal glia, resulting in the release of proinflammatory cytokines with pronociceptive effects [53]. Considering the available literature data from experimental studies, it can be concluded that the CCL1/CCR8 signaling pathway plays an important role in both CNS and PNS damage; however, further clinical studies are necessary.

Notably, the role of **CCL18**, the second endogenous CCR8 ligand present only in humans, after both CNS and PNS damage has not been determined, and clinical research on the role of the CCL18/CCR8 axis is needed.

### 9.2. CCR8—Pharmacological Modulation

Studies on the impact of the pharmacological CCR8 blockade on changes following CNS and PNS damage have yet to be conducted. These studies have only recently become possible because the first pharmacological tools, for example, AZ084 and R243, have only now been synthesized. Importantly, the results obtained after intrathecal administration of CCR8 siRNA are promising, and it has been shown that such a receptor blockade attenuates CCI-induced hypersensitivity [53]. However, in the case of damage both to the CNS and PNS, pharmacological studies with newly synthesized antagonists are still needed.

In summary, the above literature data suggest that in mice/rats/humans, the level of the CCR8 agonist CCL1 increases after PNS/CNS injury. Therefore, the CCL1-CCR8 axis seems to play an important role in neuroimmune disorders and may cause secondary CNS damage and/or the development of neuropathic pain. Clinical studies in particular are needed.

## 10. CCR9—Ligands and Pharmacological Modulation

CCR9 has only one ligand, CCL25 (please see Table 1) [393,394]. The receptor is expressed on dendritic cells, neutrophils, lymphocytes, monocyte macrophages, and vascular endothelial cells [395,396,397]. CCR9 levels are diminished in hippocampal neurons during pilocarpine-induced status epilepticus in mice [365]. The observed changes in the level of CCR9 during epilepsy suggested that this receptor may play an important, although still unknown, role in its pathomechanism, as well as in changes following CNS and PNS damage with various etiologies, and further research is certainly needed. Recently, it was found that the CCR9 gene encodes two isoforms: CCR9A and CCR9B. CCR9A most likely contains 12 additional AAs at its N-terminus. The longer N-terminal region of CCR9A may produce a receptor with enhanced affinity for CCL25. CCR9A and B are not functionally equivalent to each other. CCR9A signaling is suppressed, and CCR9A is desensitized at lower concentrations of CCL25 compared to CCR9B [398]. Yu et al. explained this phenomenon by the possibility that the active conformation of the receptor is the target of kinases that inactivate G protein-coupled receptors [399]. The decreased affinity of CCR9B for this ligand may also explain the relative insensitivity of this receptor subtype to homologous desensitization. The authors observed that, in the examined lymphocytes, there was a ∼10:1 ratio of CCR9A:CCR9B mRNA [399]. Additionally, with the use of calcium flux assays, the authors indicated that CCR9A is the most efficient receptor. To date, CCR9A and CCR9B—two CCR9 gene products resulting from differential mRNA splicing and different translation starting points—have been described in humans but not in mice [400]. Importantly, the CCR9 antagonist CCX282-B is an equally potent antagonist of mouse, rat, and human CCR9 [400]; however, the importance of the two isoforms, CCR9A and CCR9B, in the CNS and PNS needs to be studied in patients.

### 10.1. CCR9—Endogenous Ligands

**CCL25** was discovered in 1999 [393,394]; therefore, the role of the CCL25/CCR9 signaling pathway in neuroimmune changes caused by damage to the PNS and CNS should be studied. The murine recombinant CCL25 protein exhibited chemotactic effects on activated macrophages, dendritic cells, and thymocytes [401]. Literature data thus far indicate that the CCR9/CCL25 axis participates in several inflammatory diseases, including cardiovascular diseases, autoimmune diseases, hepatitis, rheumatoid arthritis, inflammatory bowel disease, and asthma [401,402], but the mechanism of action still needs to be elucidated. Moreover, it was first published in 2022 that the level of CCL25 is increased in the cerebrospinal fluid of patients who developed neuropathic pain after breast cancer surgery, which suggests that CCL25 has pronociceptive effects [361]. However, the proinflammatory and pronociceptive roles of the CCL25/CCR9 axis need to be investigated.

### 10.2. CCR9—Pharmacological Modulation

CCX282-B is a selective CCR9 antagonist and it is bioavailable in the circulation after oral administration, as assessed in mouse models of IBD and in clinical trials [401].

In summary, further research is needed to investigate the possible proinflammatory and pronociceptive effects of the CCL25-CCR9 axis after PNS/CNS injury.

## 11. CCR10—Ligands and Pharmacological Modulation

**CCR10** is a target of three chemokines, **CCL26**, **CCL27,** and **CCL28** (please see Table 1). To date, little is known about the role of this receptor after CNS/PNS injury. However, the presence of CCR10 in mouse CNS tissue at both the protein and mRNA levels has already been documented [365]. In the spinal cord (but not in the thalamus) of diabetic monkeys, astroglia and microglia cells were shown to be activated. In parallel, an increased level of CCR10 and a decrease in opioid receptors (MOP, KOP, and DOP) was observed [314]. CCR10 is also expressed in the hippocampus by neurons, in the CA1-3 region by interneurons, and in the dentate gyrus by hilar cells [365]. Moreover, GCA+ immune cells derived from bone marrow were shown to migrate to the mouse brain through the CCR10-CCL28 axis. This pathway seems to be critical for the progression of Alzheimer’s disease [403]. Importantly, CCR10 levels are reduced in hippocampal neurons during pilocarpine-induced status epilepticus in mice [365]. Moreover, CCR10 is highly expressed in human glioblastoma brain tissue [404]; therefore, this receptor seems to be essential for glioma proliferation and invasion [404]. Furthermore, an enhanced level of CCR10 has recently been associated with poor survival in glioblastoma patients [404]. In the case of hepatocarcinogenesis, the expression of CCR10 seems to act as a link between TNFα stimulation and the activation of the PI3K/Akt pathway [405]. However, the significance of CCR10 for neuroinflammation and nociceptive transmission remains to be elucidated.

### 11.1. CCR10—Endogenous Ligands

To our knowledge, there is no available information showing **CCL26**, **CCL27,** and **CCL28**’s potential role after PNS/CNS damage; therefore, both experimental and clinical studies are needed. Overall, it is only known that the CCL27/CCR10 axis can mediate glioma cell proliferation and invasion [404] and is involved in T-cell-mediated skin inflammation [406].

### 11.2. CCR10—Pharmacological Modulation

A key role of CCR10 in carcinogenesis has been demonstrated. Therefore, in the future, the blocking of this receptor may become the basis of pharmacotherapy for malignant glioma in the brain. However, there is no information about the involvement of this receptor in changes following brain, spinal, or peripheral nerve injury [404]. Recently, it was suggested that blocking CCR10 and CCR4 simultaneously is beneficial in the treatment of T-cell-mediated skin diseases [407].

In summary, the possible proinflammatory and pronociceptive roles of the CCL26/27/28-CCR10 axis after PNS/CNS injury still need to be studied.

## 12. Analgesic Potential of Dual Chemokine CC Receptor Antagonists

The results of the latest research confirmed the advantage of compounds targeting more than one molecular target over the physical combination of individual antagonists in terms of their anti-inflammatory properties, which is also the case with double CC chemokine receptor antagonists, although this type of research is still insufficient.

A small-molecule chemokine receptor antagonist, UCB 35625, which is a potent, selective blocker of CCR1 and CCR3, is an interesting double CC chemokine receptor antagonist [408]. Importantly, among the described chemokines that play important roles in secondary changes occurring after CNS and PNS damage in patients, seven CCR1 ligands (CCL2, CCL3, CCL4, CCL5, CCL7, CCL8, and CCL23) and four CCR3 ligands (CCL5, CCL7, CCL8, and CCL11) occur in humans. Notably, the abovementioned receptors share three common ligands in humans (CCL5, CCL7, and CCL8) and four common ligands in rodents (CCL5, CCL7, CCL8, and CCL9). In 2022, UCB35625 was shown to diminish hypersensitivity to thermal and mechanical stimuli in a mouse CCI model [65]. However, the analgesic effect of this dual antagonist was similar to that obtained after administrations of a single CCR1 (J113863) and CCR3 (SB328437) antagonist [65]. These results are intriguing, and further in-depth research and possibly the creation of new dual CCR1/3 antagonists are needed. As the authors suggest, the effect may depend on the strength of the substance’s binding to the receptor, but it may also depend on effectiveness, which reflects the ability of the substance to activate the receptor and generate a cellular response [65]. The efficacy of these drugs may also depend on the pharmacokinetics and pharmacodynamics of these particular compounds and not necessarily on the spectrum of their action, which is why further study on the phenomenon of the double blockade of chemokine receptors is necessary. Additionally, it is important to understand the chemical structure of antagonists and their receptors, as they may affect the obtained pharmacological response [65]. However, the effect of UCB 35625 or new dual antagonists on secondary damage after CNS damage has yet to be determined in various animal models.

Another very interesting substance is cenicriviroc, a potent selective CCR2 and CCR5 receptor antagonist. Chemokines acting through these receptors play important roles in secondary changes occurring after CNS and PNS damage in patients. There are four CCR2 ligands (CCL2, CCL7, CCL8, and CCL12) and six CCR5 ligands (CCL3, CCL4, CCL5, CCL7, CCL8, and CCL11). Two of these chemokines (CCL7 and CCL8) are common ligands of CCR2 and CCR5 in humans and rodents; for example, CCR1 and CCR3 may or may not be beneficial for the effects of dual CCR2/CCR5 antagonists. Furthermore, importantly, CCR2 and CCR5 have been shown to heterodimerize after costimulation with their ligands [409]. There are currently three dual CCR2/CCR5 antagonists available: cenicriviroc [70,85], BMS-813160 [410], and PF-04634817 [411]. However, to our knowledge, only cenicriviroc has been used in experimental animal studies thus far. In 2016, it was shown that the response of the aging brain to TBI is the increased expression of chemokines (CCL2, CCL7, CCL8, and CCL5) involved in the recruitment of peripheral macrophages to the damaged parenchyma. Importantly, the administration of cenicriviroc significantly attenuated the influx of these cells while reducing inflammatory and neurotoxic symptoms [203]. Similar beneficial effects were obtained after repeated administrations of cenicriviroc in animal models of neuropathy [70,85]. Studies after PNS damage have shown that repeated intrathecal administrations of both selective CCR2 (RS504393) [48] and CCR5 (maraviroc) [71] antagonists and a double CCR2/CCR5 antagonist (cenicriviroc) [70,85] provide pain relief. However, cenicriviroc was the most effective compound for silencing immunological changes in a rat model of CCI. After administration of cenicriviroc, the levels of six chemokines (CCL2, CCL3, CCL4, CCL5, CCL7, and CCL12), which are CCR2 and/or CCR5 ligands, were reduced in the spinal cord and/or DRG [85]. Importantly, a single intrathecal and intraperitoneal administration of cenicriviroc had been shown to have greater analgesic effects than RS504393 or maraviroc in CCI-exposed mice. Moreover, a single intraperitoneal administration of cenicriviroc enhanced the analgesic effects evoked by opioid drugs (morphine and buprenorphine) [85], which may be crucial in the context of potential clinical use. It is still unclear what underlies this beneficial phenomenon. It may be the previously described heterologous desensitization occurring between CCR2 and CCR5 [409], the already proved suppression of immunological changes caused by injury to the nervous system [85], or perhaps both. Given that cenicriviroc is presently in the final phase of clinical trials for adult HIV infection and nonalcoholic steatohepatitis treatment [412], its anti-inflammatory and analgesic properties seem to be very promising.

In summary, increasing evidence confirms the excellent analgesic efficacy of multifunctional compounds in animal models and indicates the advantage of compounds targeting more than one molecular target. The benefits for future therapy of this approach may result from simultaneous access to two or more receptors at the same dose of a pharmacologically active compound. However, further pharmacological studies are necessary.

## 13. Conclusions

Despite significant progress in modern medicine and pharmacology, nervous system diseases still pose a challenge to doctors and scientists. Numerous studies indicate that CNS/PNS damage leads to neuroimmune changes that may ultimately result in both secondary damage and pain hypersensitivity. Importantly, the analysis of the results of various studies indicates that the mechanisms occurring both at the level of the brain after direct injury and at the level of the spinal cord after nerve damage largely have a common immunological basis. Notably, chemokines and their receptors are very conserved between species, which permits the use of rodent models to conduct pharmacological experiments and draw conclusions. Understanding the role of individual chemokines from the CC family may lead to the development of innovative and effective pharmacotherapies.

**Scheme 3:**
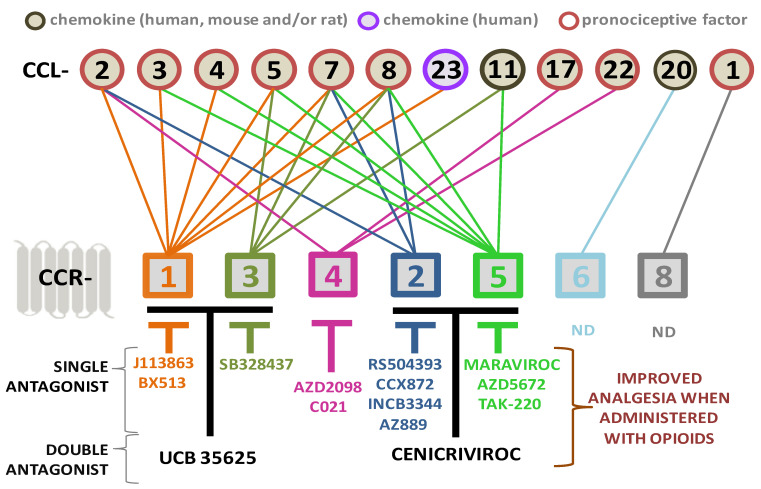
The beneficial effects of blocking the action of CC family chemokine receptors with antagonists after CNS and/or PNS damage. Additionally, the chemokines whose algesic effects have been confirmed in behavioral studies in mice/rats are marked with a red border. Scheme created based on data from the following literature [39,40,41,48,53,65,66,70,71,72,73,80,85,86,89,90,92,100,132,139,140,141,142,143,144,145,146,147,148,149,150,151,152,153,164,203,222,223,224,225,226,227,229,230,231,232,233,234,235,236,237,238,315].

Experimental data indicate that after both CNS and PNS damage, the levels of 12 of 28 chemokines from the CC family, i.e., CCL1, CCL2, CCL3, CCL4, CCL5, CCL7, CCL8, CCL9, CCL11, CCL12, CCL17, CCL20, and CCL22, increase in the brain and/or spinal cord (Table 1), and their strong proinflammatory and/or pronociceptive properties have been proven (Scheme 3).

To understand the role of the next four chemokines, i.e., CCL6, CCL8, CCL19, and CCL21, experimental research is still needed; however, preliminary results indicate changes in these cytokines after CNS and/or PNS damage (Table 1). We would like to point out that little is known about the changes and roles of the remaining five chemokines common in rodents and humans (CCL24, CCL25, CCL26, CCL27, and CCL28), as well as another six chemokines found exclusively in humans, e.g., CCL13, CCL14, CCL15, CCL16, CCL18, and CCL23 (Table 1). In the diagram, we have summarized the data about chemokines that exist in humans and their receptors that are known to play important roles in the brain and/or spinal cord after CNS and/or PNS damage (Scheme 3). Numerous behavioral studies have indicated that chemokine receptor antagonists may play an important role in therapy in the future. Available pharmacological tools indicate that blocking individual receptors, e.g., CCR1 (J113863 and BX513), CCR2 (RS504393, CCX872, INCB3344, and AZ889), CCR3 (SB328437), CCR4 (C021 and AZD-2098), and CCR5 (Maraviroc, AZD-5672, and TAK-220), has beneficial effects after both CNS and PNS damage (Scheme 3). However, it is still necessary to test whether and how blocking CCR6 and CCR8 affects the activities of their endogenous ligands, which are known to be upregulated, as is well documented for CCL1 and CCL20 after nerve and brain injury, respectively (Table 1).

Importantly, several chemokines, e.g., CCL1 [5], CCL2 [12], CCL3 [17], CCL7 [12], and CCL9 [17], decreased opioid analgesic properties in chronic pain, and the use of neutralizing antibodies restores the effectiveness of morphine and/or buprenorphine. Moreover, single (J113863 [41,66], RS504393 [48], SB328437 [80], C021 [92,230,315], and maraviroc [71,85]) and dual (cenicriviroc [70,85]) antagonists also enhance opioid analgesia. A multidirectional strategy based on the modulation of neuronal–glial–immune interactions by changing the activity of the chemokine family can significantly improve the quality of life of patients suffering from CNS and PNS damage.

Literature data indicated that novel CC-family receptor antagonists are currently being tested in patients for the management of various diseases. For example, several CCR1 antagonists have entered clinical trials, including CP-481,715 (Pfizer, New York, NY, USA) and MLN3897 (Millennium, New York, NY, USA) in rheumatoid arthritis trials, BX471 (Berlex/Scherring AG, Bothell, WA, USA) in multiple sclerosis, AZD-4818 (Astra-Zeneca, Cambridge, UK) in chronic obstructive disease spit, and BAY86-5047 for endometriosis [220,221]. The lack of beneficial effects observed in the studies in the case of some of them may be caused by their pharmacokinetic properties. For example, the CP481,715 is a competitive CCR1 antagonist; therefore, the IC_50_ varies depending on the concentration of several ligands and it can be hypothesized that their potentially high levels exceed those achieved by the drug. In this case, non-competitive antagonists would have the advantage of having activity regardless of the concentration of ligands present [220]. Another possibility of CCR1 antagonist failure can be the pleiotropic properties of its ligands, as we have shown in Scheme 3. However, the effectiveness of the next discussed compound gave far more promising results in clinical studies. The CCR5 antagonist maraviroc is an antiviral drug. By selectively binding to the CCR5 receptor, maraviroc prevents CCR5-tropic entering the cell by HIV-1 viruses [413]. However, in animal studies after CNS injury, it has been shown that maraviroc produces neuroprotective effects in TBI [232,233], FCS [234], CIBI [235], and ICH [236] models. Moreover in neuropathy models, it is able not only to diminish pain but enhance opioid effectiveness [71,85,237]. Importantly, maraviroc has received approval from the Food and Drug Administration and is used as a treatment for patients infected with human immunodeficiency virus R5 type 1 [352,353]. The fact that it is a drug used in clinics strongly suggests that it is worth trying to use it in the treatment of various diseases. Considering the good results obtained in animals, we hope that clinical trials will be conducted in patients after CNS and PNS injuries.

However, the blocking of one type of CC receptor may not be sufficient because of the overall pleiotropy and redundancy in the chemokine family. First, most of the receptors bind more than one chemokine, and second, most of the chemokines bind more than one receptor (Scheme 3), which makes planning pharmacological treatment quite a challenge. The results of the latest experimental studies confirmed the advantage of compounds targeting more than one CC chemokine receptor. It has already been proven that double antagonists, like UCB 35625 (blocker of CCR1/CCR3) [408] and cenicriviroc (blocker of CCR2/CCR5) [70,85], provide effective pain relief (Scheme 3, Table 2). Moreover, taking into account that cenicriviroc is currently in the final phase of clinical trials for adult HIV infection and nonalcoholic steatohepatitis treatment [412], its anti-inflammatory and analgesic properties seem to be very promising. Perhaps the use of a substance that would be able to block five chemokine receptors, CCR1-5 (Scheme 3), would have beneficial effects on therapy, but such an antagonist is currently unavailable.

Another approach to blocking chemokine receptor binding is the use of vMIP-II, also called viral master KEYmokine 2, vCCL2, or herpesvirus-8 macrophage inflammatory protein-II [414]. This is the only chemokine identified so far that is able to bind to six chemokine receptors from CC group (CCR1, CCR2, CCR3, CCR5, CCR8, and CCR10) but also receptors belonging to other groups (CXCR3, CXCR4, XCR1, and CX3CR1) [414,415]. vMIP-II is a chemokine encoded by human herpes virus-8 and was created by the virus to disrupt the homeostasis of the chemokine receptor network in the host and promote its survival. To date, most data on vMIP-II effects originate from in vitro studies. However, recently, it was shown that vMIP-II diminishes hypersensitivity in CCI-exposed mice [40] and improves the lymphocyte decrease in COVID-19 patients [416]. Computer tomography after a week of vMIP-II treatment showed that the lung lesions were significantly alleviated and the clinical symptoms were diminished [416]. In the light of the obtained results, it seems that the use of vMIP-II, a molecule with a broad spectrum of action, could also bring benefits after damage to the PNS and CNS, but this requires further pharmacological studies, first in animal models, and then, if good results are achieved, in clinical trials.

To sum up, in the future, the CC receptor blockers may also be used after CNS/PNS damage, but additional experimental and clinical studies are needed since the knowledge about the role of the CC family in neuroimmune changes is still insufficient. Considering the pleiotropic properties of chemokines, the pharmacological blockade of several chemokine receptors through the use of a multidirectional antagonist may be an innovative and effective method for treating CNS and PNS damage. Moreover, in the case of pain, the combined administration of multitarget CC chemokine antagonists with opioid drugs could allow for a decrease in therapeutic doses and thus minimize the risk of complications.

## Data Availability

Not applicable.
